# Supplementary material for: Pectin Digestion in Herbivorous Beetles: Impact of Pseudoenzymes Exceeds That of Their Active Counterparts
Source: Front Physiol. 2019 May 29;10:685. doi: 10.3389/fphys.2019.00685 (PMC6549527; doi:10.3389/fphys.2019.00685)
Supplement: Supplementary file 1 [file Image_1.pdf]

Supplementary Material

Pectin digestion in herbivorous beetles: Impact of pseudoenzymes exceeds that of their active counterparts

Roy Kirsch*, Grit Kunert, Heiko Vogel, Yannick Pauchet*

*** Correspondence:** Corresponding Author: rkirsch@ice.mpg.de; ypauchet@ice.mpg.de

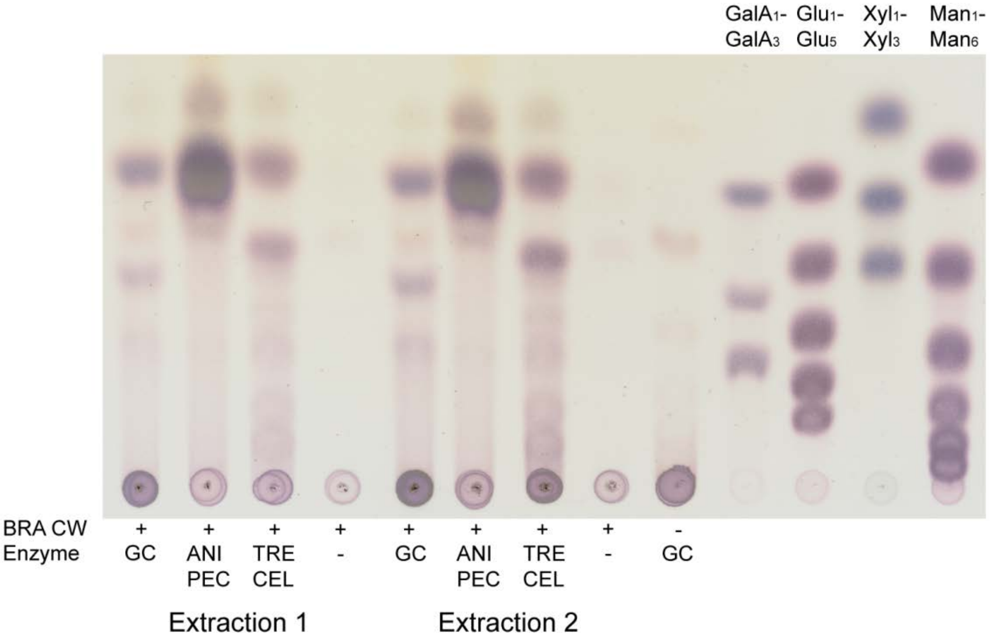

Supplementary Figure 1. Characterization of the Chinese cabbage PCW substrate. The PCW extract (BRA CW) was incubated with *Phaedon cochleariae* gut content (GC), a commercial preparation of *Aspergillus niger* pectinase (ANI PEC) and a commercial preparation of *Trichoderma reesei* cellulase (TRE CEL). Analysis of breakdown products released from the two independent preparation of PCW extracts by thin-layer-chromatography (TLC) is shown. Standards of breakdown products of the following substrates were used: pectic polygalacturonan (GalA1-3), cellulose (Glu1-5), xylan (Xyl1-3), mannan (man1-6).
