# Supplementary material for: Pectin Digestion in Herbivorous Beetles: Impact of Pseudoenzymes Exceeds That of Their Active Counterparts
Source: Front Physiol. 2019 May 29;10:685. doi: 10.3389/fphys.2019.00685 (PMC6549527; doi:10.3389/fphys.2019.00685)
Supplement: Supplementary file 2 [file Image_2.pdf]

Supplementary Material

Pectin digestion in herbivorous beetles: Impact of pseudoenzymes exceeds that of their active counterparts

Roy Kirsch*, Grit Kunert, Heiko Vogel, Yannick Pauchet*

*** Correspondence:** Corresponding Author: rkirsch@ice.mpg.de; ypauchet@ice.mpg.de

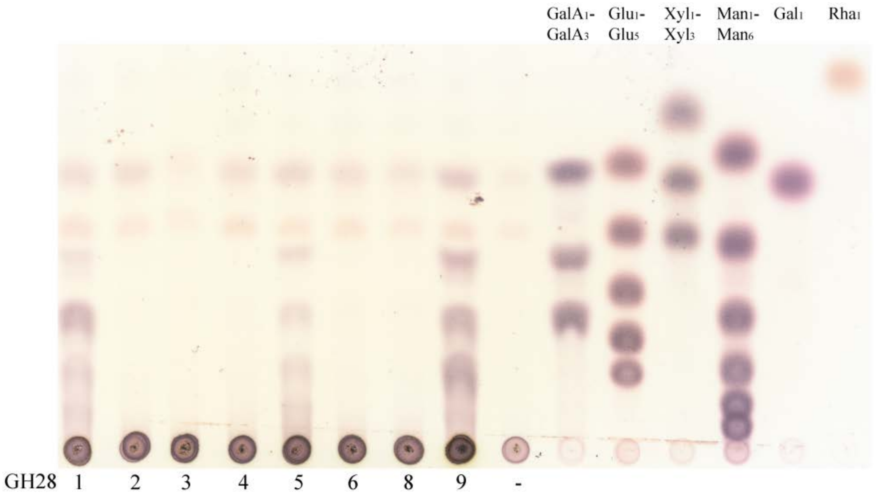

Supplementary Figure 2. Activity of *P. cochleariae* GH28s on the Chinese cabbage PCW substrate. Analysis of breakdown products of GH28s by thin-layer-chromatography (TLC) is shown. Standards of breakdown products of the following substrates were used: pectic polygalacturonan (GalA1-3), cellulose (Glu1-5), xylan (Xyl1-3), mannan (man1-6) as well as galactose (Gal) and rhamnose (Rha) monomers, which are decorations of pectin.
