# Supplementary material for: Pectin Digestion in Herbivorous Beetles: Impact of Pseudoenzymes Exceeds That of Their Active Counterparts
Source: Front Physiol. 2019 May 29;10:685. doi: 10.3389/fphys.2019.00685 (PMC6549527; doi:10.3389/fphys.2019.00685)
Supplement: Supplementary file 3 [file Image_3.pdf]

Supplementary Material

Pectin digestion in herbivorous beetles: Impact of pseudoenzymes exceeds that of their active counterparts

Roy Kirsch*, Grit Kunert, Heiko Vogel, Yannick Pauchet*

*** Correspondence:** Corresponding Author: rkirsch@ice.mpg.de; ypauchet@ice.mpg.de

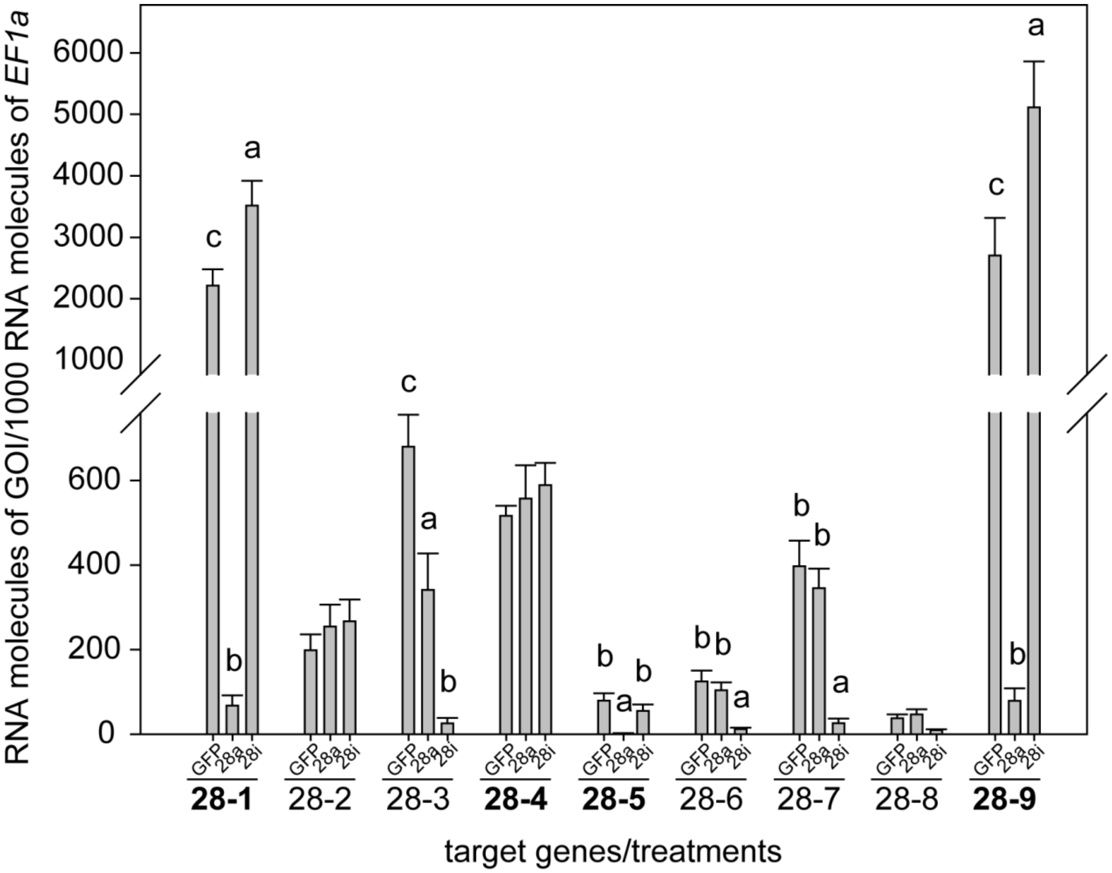

Supplementary Figure 3. General overview of the expression patterns of all *P. cochleariae* GH28s over all treatments comparing injection control (GFP), active GH28 silencing (28a) and inactive GH28 silencing (28i). Active GH28s are indicated in bold. Transcript abundances are expressed as RNA molecules of gene of interest (GOI) per 1000 RNA molecules of the reference gene elongation factor 1-alpha (*EF-1α*).
