# Supplementary material for: Pectin Digestion in Herbivorous Beetles: Impact of Pseudoenzymes Exceeds That of Their Active Counterparts
Source: Front Physiol. 2019 May 29;10:685. doi: 10.3389/fphys.2019.00685 (PMC6549527; doi:10.3389/fphys.2019.00685)
Supplement: Supplementary file 4 [file Table_1.DOCX]

Supplementary Material

Pectin digestion in herbivorous beetles: Impact of pseudoenzymes exceeds that of their active counterparts

Roy Kirsch*, Grit Kunert, Heiko Vogel, Yannick Pauchet*

*** Correspondence:** Corresponding Author: rkirsch@ice.mpg.de; ypauchet@ice.mpg.de

**Supplementary Table 1.** List of primers used in this study.

| **Primer sequence** | **Application** |
| --- | --- |
| TAATACGACTCACTATAGGGCGATCAGATTGATAGCCGTAC | forward primer for amplification of templates to prepare dsRNA targeting 28-1; T7 RNA polymerase binding motif |
| TAATACGACTCACTATAGGGCGGCTTCGTTATTCCTCCG | reverse primer for amplification of templates to prepare dsRNA targeting 28-1; T7 RNA polymerase binding motif |
| TAATACGACTCACTATAGGGGTCCTCTGATCCAGATCAGC | forward primer for amplification of templates to prepare dsRNA targeting 28-5; T7 RNA polymerase binding motif |
| TAATACGACTCACTATAGGGGAAGCCGTCGAGGGTCAC | reverse primer for amplification of templates to prepare dsRNA targeting 28-5; T7 RNA polymerase binding motif |
| TAATACGACTCACTATAGGGCGGAGTTGCGCATTGGGATG | forward primer for amplification of templates to prepare dsRNA targeting 28-9; T7 RNA polymerase binding motif |
| TAATACGACTCACTATAGGGCCAGCCGTCGATTGTCACG | reverse primer for amplification of templates to prepare dsRNA targeting 28-9; T7 RNA polymerase binding motif |
| TAATACGACTCACTATAGGGACAAGCTATCTGACGCAGC | forward primer for amplification of templates to prepare dsRNA targeting 28-3; T7 RNA polymerase binding motif |
| TAATACGACTCACTATAGGGAACAGATCCGCCTGTAGC | reverse primer for amplification of templates to prepare dsRNA targeting 28-3; T7 RNA polymerase binding motif |
| TAATACGACTCACTATAGGGTCACCTCATCCATGGACAG | forward primer for amplification of templates to prepare dsRNA targeting 28-6; T7 RNA polymerase binding motif |
| TAATACGACTCACTATAGGGATGGACATTCCTGATTACAGC | reverse primer for amplification of templates to prepare dsRNA targeting 28-6; T7 RNA polymerase binding motif |
| TAATACGACTCACTATAGGGTGGAACCAAGACGATTGCG | forward primer for amplification of templates to prepare dsRNA targeting 28-7; T7 RNA polymerase binding motif |
| TAATACGACTCACTATAGGGTCAATCGCTCTCATGATGACATC | reverse primer for amplification of templates to prepare dsRNA targeting 28-7; T7 RNA polymerase binding motif |
| TCAGTTGTTCGCAAATCGAG | forward qPCR primer 28-1 |
| CAGTGGGTTTGCCCTTCTTA | reverse qPCR primer 28-1 |
| GAGACGGAGACAACGGGATA | forward qPCR primer 28-2 |
| CATGGTTCCAGTGATGTTCG | reverse qPCR primer 28-2 |
| CCTTGGAGACAACTGCACAA | forward qPCR primer 28-3 |
| TGCGTAGTCGAAAGTGATGG | reverse qPCR primer 28-3 |
| GACGCCAACTAAAGACTGCAC | forward qPCR primer 28-4 |
| AGGTGTGTGCTCGAACAGAGT | reverse qPCR primer 28-4 |
| GTTCTCAGTGGCCTCCACATT | forward qPCR primer 28-5 |
| GGTCACGTAGTGGGCTTTGT | reverse qPCR primer 28-5 |
| GGAGATGGTAATTCGGCAAA | forward qPCR primer 28-6 |
| AGAGCACGATCCAGAACCAC | reverse qPCR primer 28-6 |
| ATGCACTCACATCGTTCTTCAG | forward qPCR primer 28-7 |
| GAGGTGATTAGTTCCTCCGATG | reverse qPCR primer 28-7 |
| GATGTCACTCAGAGAGCAATCG | forward qPCR primer 28-8 |
| ACTCCTACTGCGCTTCTACCAG | reverse qPCR primer 28-8 |
| TCGGTAGCAAGGTCACTGTC | forward qPCR primer 28-9 |
| AGACATGTAGGCCGGTCAGG | reverse qPCR primer 28-9 |
| ATGGGCTGTTGAACGTAAGG | forward qPCR primer elongation factor 1a |
| CCACACGGCCTACTGGTACT | reverse qPCR primer elongation factor 1a |
