# Supplementary material for: Pectin Digestion in Herbivorous Beetles: Impact of Pseudoenzymes Exceeds That of Their Active Counterparts
Source: Front Physiol. 2019 May 29;10:685. doi: 10.3389/fphys.2019.00685 (PMC6549527; doi:10.3389/fphys.2019.00685)
Supplement: Supplementary file 5 [file Table_2.DOCX]

Supplementary Material

Pectin digestion in herbivorous beetles: Impact of pseudoenzymes exceeds that of their active counterparts

Roy Kirsch*, Grit Kunert, Heiko Vogel, Yannick Pauchet*

*** Correspondence:** Corresponding Author: rkirsch@ice.mpg.de; ypauchet@ice.mpg.de

**Supplementary Table 2.** Differential expression (fold-change values) of the GH28 genes, and the statistical significance thereof (Student´s t-test; FDR-corrected p-values) obtained from the digital gene expression analysis (see Figure 2).

| **Description** | **Pco-ActiveGH28 vs PCO-GFP - FoldChange** | **Pco-ActiveGH28 vs PCO-GFP -P value** |
| --- | --- | --- |
| GH1-1 | 1,172 down | 1 |
| GH1-2 | 3,361 up | 0.593 |
| GH1-3 | 1,578 up | 1 |
| GH1-4 | 1,031 down | 1 |
| GH1-5 | 1,164 up | 0.992 |
| GH1-6 | 1,152 down | 0.909 |
| GH1-7 | 1,386 down | 1 |
| GH1-8 | 1,228 up | 1 |
| GH1-9 | 1,020 down | 1 |
| GH1-10 | 1,561 up | 1 |
| GH1-11 | 1,284 up | 0.909 |
| GH11-1 | 1,073 up | 1 |
| GH11-2 | 1,214 up | 1 |
| GH28-1 | 107,550 down | 0.0256 |
| GH28-2 | 1,555 up | 0.968 |
| GH28-3 | 1,557 down | 1 |
| GH28-4 | 1,083 down | 1 |
| GH28-5 | 27,618 down | 0.0257 |
| GH28-6 | 1,022 down | 1 |
| GH28-7 | 1,215 down | 1 |
| GH28-8 | 1,233 down | 1 |
| GH28-9 | 36,759 down | 0.0196 |
| GH45-1 | 1,266 up | 1 |
| GH45-2 | 1,255 up | 1 |
| GH45-3 | 1,245 up | 1 |
| GH45-4 | 1,110 up | 1 |
| GH45-5 | 1,527 up | 1 |
| GH45-6 | 2,143 up | 1 |
| GH45-7 | 1,268 up | 1 |
| GH45-8 | 1,058 up | 1 |
| GH48-1 | 1,114 up | 1 |
| GH48-2 | 1,100 up | 1 |
| RPS18 | 1.059 down | 1 |
| RPL7 | 1.114 down | 1 |
| **Description** | **Pco-InactiveGH28 vs PCO-GFP - FoldChange** | **Pco-InactiveGH28 vs PCO-GFP - P value** |
| GH1-1 | 1,178 down | 1 |
| GH1-2 | 3,114 up | 0.393 |
| GH1-3 | 1,015 down | 0.893 |
| GH1-4 | 1,132 down | 0.893 |
| GH1-5 | 1,216 up | 1 |
| GH1-6 | 1,078 down | 1 |
| GH1-7 | 1,098 up | 1 |
| GH1-8 | 1,029 up | 0.924 |
| GH1-9 | 1,235 down | 1 |
| GH1-10 | 2,311 up | 1 |
| GH1-11 | 1,060 up | 1 |
| GH11-1 | 1,097 up | 1 |
| GH11-2 | 1,232 up | 1 |
| GH28-1 | 1,426 up | 0.899 |
| GH28-2 | 1,035 up | 1 |
| GH28-3 | 62,886 down | 0.0226 |
| GH28-4 | 1,122 up | 0.987 |
| GH28-5 | 1,151 down | 1 |
| GH28-6 | 16,509 down | 0.0247 |
| GH28-7 | 82,876 down | 0.0118 |
| GH28-8 | 3,441 down | 1 |
| GH28-9 | 3,305 up | 1 |
| GH45-1 | 1,030 up | 1 |
| GH45-2 | 1,300 up | 1 |
| GH45-3 | 1,328 up | 1 |
| GH45-4 | 1,142 up | 1 |
| GH45-5 | 1,072 up | 1 |
| GH45-6 | 1,115 up | 1 |
| GH45-7 | 1,027 down | 1 |
| GH45-8 | 1,057 down | 1 |
| GH48-1 | 1,026 up | 1 |
| GH48-2 | 1,005 up | 1 |
| RPS18 | 1.137 down | 1 |
| RPL7 | 1.117 down | 1 |
